# Social Cognitive Correlates of Physical Activity among Chinese University Employees: A Cross-Sectional Study

**DOI:** 10.3390/ijerph18137116

**Published:** 2021-07-02

**Authors:** Liang Hu, Qia Hu, Yaping Xu

**Affiliations:** 1 Department of Sport and Exercise Sciences, Zhejiang University, Hangzhou 310098, China; lianghu@zju.edu.cn (L.H.); 21803038@zju.edu.cn (Q.H.); 2 Department of Physical and Art Education, Zhejiang University, Hangzhou 310098, China

**Keywords:** physical activity, university employees, self-efficacy, outcome expectation, social support, importance

## Abstract

Despite the well-documented benefits of leisure time physical activity, university employees are often reported to be at high risk of physical inactivity and low fitness levels. However, few efforts have been made to identify modifiable correlates of physical activity among this population. From the perspective of Social Cognitive Theory (SCT), the current study aims to examine the relationship between physical activity and a series of demographic variables (e.g., age, gender, income, education), self-reported fitness, and social cognitive variables. Data were collected through mail-based surveys from a convenience sample consisting of 116 Chinese university employees (age = 36.59 ± 8.7 y). An array of SCT variables, namely, exercise self-efficacy (*r* = 0.55, *p* < 0.01), barrier self-efficacy (*r* = 0.35, *p* < 0.01), exercise social support (*r* = 0.37, *p* < 0.01), importance of physical self (*r* = 0.30, *p* < 0.01), outcome expectations (*r* = 0.24, *p* < 0.05), and satisfaction with health(*r* = 0.32, *p* < 0.01) were found to be positively correlated with physical activity in Chinese faculty and staff, and most of the correlations were moderate to large in magnitude. Further regression analyses indicate that exercise self-efficacy (*β* = 0.29, *p* < 0.01) and exercise social support from friends (*β* = 0.70, *p* < 0.01) emerge as significant predictors of physical activity after controlling for age, gender, occupation (faculty or staff), and self-reported fitness. It is concluded that these SCT variables are important correlates of university employees’ physical activity behavior. Future physical activity promotion interventions in this population should incorporate strategies to improve one’s confidence in maintaining regular physical activity and enhance social support from friends, which are likely to increase the effectiveness of these programs.

## 1. Introduction

Although regular physical activity has been associated with a number of physical and psychological health benefits [1], lacking sufficient physical activity participation is still increasingly prevalent worldwide [2], and such a trend may be particularly prominent in certain professions. University employees are often associated with a highly sedentary and stressful working pattern, and thus represent a population at increased risk of physical inactivity [3,4]. Accumulating evidence suggests that lack of physical activity and low fitness level have become alarming health issues among Chinese university employees in recent decades. Based on a sample of 507 faculty and staff members at a Chinese university, a study indicated that less than 20% of the participants under 50 years engaged in regular physical exercise, which is much lower than the percentage of the general Chinese population in the same age group [5]. Likewise, a survey involving 1337 faculty members in 81 universities of Jiangsu Province, China found that only 29.87% of them engaged in over 30 min of physical exercise three times a week [6]. The prevalence of physical inactivity has been consistently confirmed by a series of studies targeting Chinese university employees including both faculty and staff [7,8]. Such an issue calls for effective lifestyle interventions from a public health perspective for the following reasons: First, physically inactive university employees are likely to suffer lower levels of fitness (e.g., cardiovascular fitness, strength, flexibility), adverse health conditions (e.g., high blood pressure, obesity) [5], job burnout, and impaired well-being [4]. Considering university employees play major roles in advancing higher education and science, the above-mentioned issues lead to not only the loss of productivity and quality of life among university employees, but also impose negative consequences on societal development. Second, university employees can serve as role model for young generations. If working at universities is associated with overtime working, striving for productivity at the price of leisure exercise, health, and well-being, such a negative image can be damaging and potentially discourage people from pursuing a career at university. Therefore, it is unsurprising that wellness programs within universities are becoming increasingly common, and promoting physical activity is a critical component of these programs [3]. In fact, the university appears to be an ideal setting for physical activity interventions given that university employees usually have good health literacy, easy access to relatively low-cost exercise facilities, and flexibility in accommodating exercise routines into their daily schedule. The abundancy of opportunities of participating physical activity and lack of actions among university employees appears to be a paradox, which represents an issue for researchers to explore.

Understanding the correlates and determinants, especially those modifiable ones, is the fundamental step towards successfully developing and implementing physical activity interventions for a target population. It has been contended that such efforts should be made in a theory-guided manner, as theory offers a justifiable framework for understanding physical activity behavior, and theory-based behavioral interventions are generally more effective than atheoretical ones [9,10]. Currently, a few studies have attempted to adopt behavioral theories to explain physical activity among university employees. Zhao [11] used the transtheoretical model [12] as a theoretical framework to categorize 600 university employees from Guangdong, China into five exercise stages, and respectively identified key correlates of physical activity (e.g., self-regulation, outcome evaluation) in each stage. However, theory-based research on physical activity correlates in the university setting has mainly focused on college students [13], and university employees remain an understudied group in this regard.

Social cognitive theory (SCT) [14] has been widely adopted in explaining, predicting, and intervening in health behaviors [15] including physical activity. The SCT framework is constructed on the basis that assume people form, and subsequently act upon, expectancies of behavioral outcomes. Such expectancies are mainly focused on behavioral outcomes (e.g., benefits/barriers, outcome expectations) and on one’s capability to carry out the course of action that points to a given outcome (e.g., self-efficacy, perceived competence), which subsequently leads to actual goal-oriented behaviors [10]. During this process, a network of socio-cognitive constructs in three domains, namely human, environment, and behavior, interact with each other in a reciprocally determining manner [16].

The application of SCT in physical behavior promotion has been proved successful by hundreds of observational and experimental studies [9,10]. Hence, when physical activity interventions are to be designed and delivered to a target population, starting with identifying social cognitive correlates of physical activity is an appealing and promising approach, as cognitive variables are believed to be the proximal factors to behavior and more open to change than other more stable factors (e.g., socio-demographic and personality factors) [9]. Such an approach has been successfully adopted by physical activity studies targeting various sub-populations such as Latinos [17], individuals with Multiple Sclerosis [18,19], and Korean male high-school students [20]. An early investigation among faculty and staff from three universities in the U.S. [21] found that maintainers of physical activity were more likely than non-maintainers to have a plan for exercise, higher levels of self-efficacy, and lower scores in excuse making, suggesting that planning and self-efficacy, as key SCT variables, are important for successfully maintaining long-term physical activity. However, the study only included two SCT variables and did not examine their contribution to physical activity. Therefore, the current study aims to adopt social cognitive theory [14] as a framework to identify SCT correlates of physical activity and examine their contribution to physical activity among Chinese university employees. We hypothesized that a set of core SCT variables (self-efficacy, outcome expectations, social support, physical activity importance, and health satisfaction) would emerge as significant correlates of physical activity behavior, and thus could potentially serve as the targets of physical activity promotion programs for Chinese university employees.

## 2. Methods

### 2.1. Procedures

This study was approved by a university institutional review board. Participants were recruited from four universities in three provinces of China (i.e., Zhejiang, Gansu, and Guangxi) through social media and referral. Once individuals expressed interest in participating in the study, the research team then contacted the potential participants by phone or email, and mailed them a package including two copies of an informed consent form (one for them to sign and return, and the other for them to keep as record) and a package of questionnaires. The participants were asked to return the signed inform consent form and questionnaires within 7 days upon receiving them. To minimize the influence of social desirability, the participants answered the survey anonymously. In addition, an instruction paragraph was included on the first page of the survey to ensure the participants that their identity would not be revealed or disclosed, and they could simply answer the surveys that best describe their situation. In total, 120 participants agreed to participate in the study and 116 of them returned the questionnaires. Thirty Chinese dollars were paid in appreciation for their participation in the study.

### 2.2. Measures

#### 2.2.1. Demographic Information

Each participant was asked to provide their age, gender (1 = Male, 2 = Female), education (1 = High School and Below, 2 = College, 3 = Master, 4 = PhD), and monthly income (1 = Less than ¥1000, 2 = ¥1000–4999, 3 = ¥5000–9999, 4 = ¥10,000–19,999, 5= Over ¥20,000), as well as marital status (1 = Married, 2 = Single/Divorced, 3 = Others), smoking status (1 = Frequently, 2 = Sometimes, 3 = Rarely, 4 = Do not smoke), occupational status (1 = Faculty, 2 = Staff), and number of children.

#### 2.2.2. Physical Activity

Physical activity was measured by the Godin Leisure-Time Exercise Questionnaire (GLTEQ) [22]. The GLTEQ includes 3 open-ended questions that measure the frequency of exercise sessions over 15 min performed at strenuous, moderate, and mild intensity respectively during one’s free time in the preceding week. The weekly frequencies of strenuous, moderate, and mild activities are multiplied by 9, 5, and 3 metabolic equivalents, respectively, and the sum is calculated to form a measure of total leisure activity. The GLTEQ was adopted for its practicality and ease of administration, and it has demonstrated adequate validity in both general adult population and academic professors [23].

#### 2.2.3. Self-Reported Physical Fitness

The Physical Fitness Questionnaire (FFB-Mot) [24] was utilized to measure motor fitness status in a self-reported manner. The standard FFB-Mot questionnaire comprises 20 self-reported items assessing four basic motor abilities, namely cardiorespiratory fitness, strength, flexibility, and coordination. For each item (e.g., Can you complete two kilometers brisk walking without stopping), the participants rated it on a Likert-point scale (1 = No problem at all, 5 = Unable to complete at all). The total score is generated by summing ratings of all items, with lower scores indicating higher physical fitness. High correlations between this self-report tool of physical fitness and physical fitness tests were supported by validation studies. The FFB-Mot has been applied and validated as an economical screening method for the assessment of physical fitness in adult populations [25].

### 2.3. SCT Variables

#### 2.3.1. Exercise Self-Efficacy

The 6-item Exercise Self-Efficacy scale [26]. This scale assesses one’s belief in his or her capability to exercise three times a week at a moderate intensity. The first item presents a statement “*I am able to continue to exercise three times per week at moderate intensity, for 40+ minutes without quitting for the NEXT MONTH*”, whereas each of the other five items represents an increment of one month in duration, ranging from 2 to 6 months. Using a 100-point percentage scale comprising 10-point increments (1 = no confidence at all, 10 = complete confidence), the participants indicated their confidence in their ability to exercise for each item. The self-efficacy score was calculated by dividing the total of the confidence ratings by the number of items in the scale, resulting in a total score with a possible range of 0 to 10. McAuley et al. [22] reported that internal consistency for the measure was acceptable (*α* = 0.85) and the measure was found to be predictive of exercise adherence [26]. The internal consistency for this scale in this study was excellent (*α* = 0.97).

#### 2.3.2. Barrier Self-Efficacy

The Barrier Self-Efficacy scale [27] was employed to measure participants’ perceived abilities to exercise 3 times per week for 40 min in the face of commonly identified barriers to participation. For each item, the participants indicate their confidence about executing the behavior on a 100-point percentage scale composed of 10-point increments ranging from 0% (not at all confident) to 100% (highly confident). Total strength for this measure of self-efficacy is then calculated by summing the confidence ratings and dividing by the total number of items in the scale, resulting in a maximum possible efficacy score of 100. McAuley et al. [27] provided support for its good reliability (*α* = 0.77 to 0.88) and construct validity in the item factor analysis. Internal consistency for this scale in the current study was good (*α* = 0.82).

#### 2.3.3. Exercise Social Support

The Social Support for Exercise scale (SSE) [28] is a 10-item scale assessing the degree to which friends and family have demonstrated verbal and behavioral support for exercise behaviors in the previous 3 months. The frequency for each of the 10 items is rated twice on a 5-point Likert scale, ranging from 1 (never) to 5 (very often), once for family members, and once for friends. Example items include “exercised with me,” and “gave me helpful reminders to exercise”. In the study that developed the SSE [24], this scale not only demonstrated test-retest and internal consistency reliability, but the factor structure and criterion-related validity were also supported. In the current study, the subscale for friend support (*α* = 0.91) and family support (*α* = 0.92) both demonstrated excellent internal consistency.

#### 2.3.4. Outcome Expectations for Exercise

The Outcome Expectations for Exercise scale (OEE) [29] is a 9-item scale that was developed based on several previously tested measures that focused on the outcome expectations and benefits associated with exercise in adults. To complete the OEE scale the participants were asked to read a statement about exercise (for example, “Exercise makes me feel better physically”) and to strongly agree (1), agree (2), neither agree nor disagree (3), disagree (4), or strongly disagree (5) with the stated outcomes or benefits of exercising. The scale is scored by summing the numerical ratings for each response and dividing by the number of responses. Lee et al. [30] validated the Chinese version of OEE was by demonstrating that this scale possesses acceptable internal consistency (alpha = 0.85) and model fit. In addition, an analysis of the Mokken Scaling Procedure found that nine items of the scale were all retained in the analysis and the resulting scale was reliable and statistically significant. Excellent internal consistency was demonstrated for this scale in this study (*α* = 0.90).

#### 2.3.5. Importance of Physical Self

The Perceived Importance Profile was employed to determine the degree to which participants value their physical self, which is divided into four subdomains: physical condition, an attractive body, and physical strength [31]. Each of the value ratings is comprised of two items with item scores ranging from 1 (not at all true) to 4 (completely true). This scale is administered as a part of the Physical Self-Perception Profile (PSPP), an instrument assessing self-esteem relative to the physical domain, and the factor structure was also supported in the validation study of PSPP [31]. The six items are combined to form an importance index of physical self. Excellent internal consistency was demonstrated for this scale in this study (*α* = 0.90).

#### 2.3.6. Health Satisfaction

Health satisfaction was assessed by the subscales of Life Satisfaction scales [32]. This scale evaluated the degree of satisfaction about physical health, including 7 items (e.g., Are you satisfied with your health status?), rating on 7-point scales (1 = absolutely dissatisfied; 2 = somewhat dissatisfied; 3 = little dissatisfied; 4 = neither dissatisfied nor satisfied; 5 = little satisfied; 6 = somewhat satisfied; 7 = absolutely satisfied). The higher scores represent the higher health satisfaction. Fahrenberg et al. [32] found that this scale has good construct validity and internal consistency in their development study of the Life Satisfaction scales. Cronbach’s alpha in the current study was high (*α* = 0.90).

All of the questionnaires were translated into Chinese and then back-translated from Chinese into English following the procedure adopted by Chan et al. [33].

### 2.4. Statistics Analysis

All analyses were conducted using IBM Statistics, version 24.0 (IBM Corp, Armonk, NY, USA) in two steps: (1) Correlational analyses were conducted to determine whether demographic variables, self-reported fitness level, and SCT factors were associated with physical activity. (2) Based on the correlational analyses, the SCT variables that were associated with physical activity levels were entered to hierarchical regression analyses to examine whether or not they uniquely account for the variance in physical activity controlling for demographic covariates (i.e., gender, age, occupation, marital status, number of children, education, income, and smoking status) and self-reported fitness level.

## 3. Results

### 3.1. Sample Characteristics

A convenience sample of 120 participants was recruited and returned the questionnaires. After removing four questionnaires that were barely answered, 116 participants were included in the final analyses. The participants were mostly female and married, aged between 24 and 59 years, with a mean age of 37. The descriptive characteristics of the sample are provided in Table 1.

### 3.2. Correlations of Physical Activity and Demographic Variables, Self-Reported Fitness, and SCT Variables

Occupation (1 = faculty, 2 = staff; *r* = −0.24, *p* < 0.05), but none of the other demographic variables (age, gender, marital status, number of children, education, income, smoking status, and frequency of staying up), was significantly correlated with physical activity. A higher level of physical activity was associated with higher self-reported fitness levels in terms of both subscales and total score.

The correlations between physical activity and SCT variables for faculty and staff participants and the total sample are shown in Table 2. Physical activity expressed by Godin scores were significantly and positively associated with all SCT variables, i.e., exercise self-efficacy, barrier self-efficacy, physical activity importance, exercise social support (for friends, family subscales, and total score), outcome expectation for exercise, and health satisfaction, and most of the correlations were moderate in magnitude (*rs* = 0.23–0.55; all *p* values < 0.05) based on Cohen’s guideline on determining effect size of correlation coefficient [34].

### 3.3. Predicting Physical Activity from Demographic Variables, Self-Reported Physical Fitness, and SCT Correlates

To evaluate the unique contribution of demographic variables, self-reported physical fitness, and SCT correlates to the variance of physical activity, a set of hierarchical regression analyses were conducted in a stepwise manner, and these variables were entered as three ordered sets of predictors in the prediction models of physical activity. In Step 1, age, gender, and occupation were entered in as Model 1; in Step 2, self-reported fitness (total) was entered in addition to age, gender, and occupation to formulate Model 2; in Step 3, all the SCT variables were further entered to formulate Model 3.

The results (see Table 3) suggest that exercise self-efficacy (*β* = 0.39, *p* < 0.01) and social support for exercise from friends (*β* = 0.27, *p* < 0.01) significantly predict physical activity after controlling for other significant correlates of physical activity (i.e., gender, occupation, and self-reported fitness).

## 4. Discussion

Based on a convenience sample of Chinese university employees, the current study identified an array of SCT correlates of physical activity in this group. It appears that those were more confident in their capabilities in performing exercising and continuing to do so in the face of barriers received more support for exercising from family and friends, put more value on their physical self, and had higher satisfaction with their health status are more likely to be physically active. Although previous evidence exists to suggest that the levels of some social cognitive variables (exercise self-efficacy and planning) were higher among physical activity maintainers than non-maintainers [21], to our knowledge, no studies have attempted to identify a set of social cognitive correlates and examine their contribution to physical activity among university employees. Our study addressed this research gap and lends support for the veracity of applying the SCT framework in the promotion of physical activity among the university working population.

During the past 40 years of reform and opening up in China, a major transformation of Chinese higher education has taken place. Accordingly, the rapid development, expansion of university student enrollment, and elevated standards of teacher recruitment due to more available well-trained candidates nowadays have not only advanced the quality of education, but also put university employees under substantial pressure. The increasing workload of teaching and research, together with the commonly observed sedentary working pattern [3] and physically inactive lifestyle [7] were considered substantial causes of mental health issues [4] and a number of other health issues in this group. Although university employees’ physical fitness is usually at the same level with that of the general population [35], there is evidence to show that they are at high risk of being overweight and obese, have cardiovascular risk, and have low levels of flexibility, balance, and strength [8], and such health issues were particularly prevalent among those over 45 years [36]. University employees are often overwhelmed by excessive job demands, which may create a stronger sense of time inadequacy. Reports indicate that Chinese university employees are likely to work overtime outside of regular office hours. Although they intend to exercise, sense of lacking time remains a consistently perceived barrier to physical activity [7].

It is therefore unsurprising that in recent decades, increasing research attention has been directed towards the issues of insufficient physical activity participation and accompanying health consequences (e.g., reduced fitness and increased risk of developing chronic disease) among Chinese university employees [8,35,36]. The need for designing and implementing physical activity promotion programs for this population has been voiced out. However, as a fundamental step toward this goal, exploring malleable correlates of physical activity in this population remains understudied.

The current study, as expected, found that SCT variables such as exercise self-efficacy, barrier self-efficacy, exercise social support, importance of physical self, outcome expectations, and satisfaction with health were all positively correlated with physical activity. Importantly, the magnitudes of most associations were moderate or large according to the Cohen’s criteria of effect size for correlations [34], with social support provide by family members being the only factor that had a small correlation with physical activity. It should be particularly noted that among an array of correlates, the exercise self-efficacy and familial support for exercise independently contributed to physical activity above and beyond the influence of perceived fitness, occupation, and other SCT variables. Our findings are in line with a number of previous endeavors that confirmed the correlations between SCT constructs in various sub-populations such as adolescents [37], individuals with depression [38] and Multiple Sclerosis [18], and Latinos [17].

According to Bandura [14,15,16], SCT variables are malleable using psychosocial strategies. To this end, our findings should be interpreted in the context of health promotion as they lend support for applying the SCT in understanding, and potentially intervening in physical activity behaviors among Chinese university employees. The SCT not only offered a theoretical framework for understanding the influence of socio-cognitive constructs on human’s health behavior, but also provided operational guidance on how to shape an individual’s cognitive thinking and direct his/her goal-oriented behavior [13]. For instance, one’s efficacy beliefs can be manipulated by strengthening mastery experience and vicarious experience, providing verbal persuasion, and appropriately interpreting physiological and cognitive state. Likewise, social support can be elicited by informing family members the importance of physical self, as well as principles and practical means of behavioral management [15]. Indeed, the veracity of designing physical activity interventions by targeting SCT variables has been confirmed among both adolescents [39,40] and adults [41]. Most university employees typically have the advantages of being well-educated, possessing high levels of health literacy, and having inexpensive access to on-campus exercise facilities, making them ideal target for SCT-based physical activity promotion programs. Although exercise regimens are often recommended to university employees and most of them have the intention to be physically active [7], without successfully addressing the intention-behavior gap with effective behavioral strategies, efforts of promoting physical activity are likely to be fruitless. In this regard, enhancing the influential psychosocial factors (i.e., self-efficacy, exercise social support, value of importance of physical self, satisfaction with health) may be of essential importance to effectively implementing exercise programs for Chinese university employees.

This study was certainly not without limitations. First, the cross-sectional nature of the study is appropriate for generating informative research knowledge but excluded the possibility of inferring causality. Considering SCT proposes a reciprocal determinism between personal, environmental, and behavioral factors, future studies are warranted to examine the interplay between SCT variables and physical activity in a bi-directional manner. Second, the data collection relied on self-report, so the studies should be replicated and strengthened by the inclusion of objective measures (e.g., accelerometers and pedometers) of physical activity and fitness level. Third, the participants were limited to three provinces in China and the sample size was relatively small. This may limit the statistical power of our analyses and may explain the insignificant correlations between physical activity and several SCT variables in this group because the magnitude of several insignificant correlations was still moderate. Future endeavors may seek to recruit a nation-wide sample of large size and extend the study to other professions of similar nature (e.g., predominantly sedentary working pattern, high job stress), in an effort to increase the generalizability of study findings.

## 5. Conclusions

We provide support for an array of SCT correlates of physical activity among Chinese university employees. Future behavioral interventions should seek effective strategies to enhance these variables, especially exercise self-efficacy and social support, which have the potential to increase physical activity participation in this population.

## Figures and Tables

**Table 1:** Characteristics of participants (n = 116).

	Faculty (n = 83)	Staff (n = 23)	Total (n = 116)	*χ^2^*
Age range	24–59	25–56	24–59	
Age (M + SD)	35.8 ± 7.9	39.4 ± 10.8	36.59 ± 8.7	
Gender				
male	51 (55.8%)	4 (17.4%)	55 (47.7%)	12.61 **
female	40 (44.2%)	21 (82.6%)	61 (52.3%)	
Marital Status				
Married	75 (82.4%)	19 (76.0%)	94 (81.0%)	3.80
Single/Divorced	16 (17.6%)	5 (20.0%)	21 (18.1%)	
Other	0 (0%)	1 (4.0%)	1 (0.9%)	
Number of children				
0	36 (39.6%)	9 (36%)	45 (38.8%)	0.149
1	47 (51.6%)	14 (56%)	61 (52.6%)	
2 and more	8 (8.8%)	2 (8%)	10 (8.6%)	
Education Level				
Undergraduate and below	2 (2.2%)	2 (8%)	4 (3.5%)	15.21 **
Master degree	25 (27.8%)	16 (64%)	41 (35.7%)	
Doctoral degree	56 (62.2%)	7 (28%)	63 (54.8%)	
Monthly Income				
Less than 1000 Yuan	0 (0%)	0 (0%)	0 (0%)	2.84
1000–4999 Yuan	46 (50.5%)	17 (68%)	63 (54.3%)	
5000–9999 Yuan	42 (46.2%)	8 (32%)	50 (43.1%)	
10,000–20,000 Yuan	1 (1.1%)	0 (0%)	1 (0.9%)	
More than 20,000 Yuan	2 (2.2%)	0 (0%)	2 (1.7%)	

Note: Chi-square tests were performed to examine if significant difference exists in the distribution in the gender, marital status, number of children, education level, and monthly income variables by occupation (faculty vs. staff); ** = *p* < 0.01.

**Table 2:** Correlations of physical activity, demographic variables, and SCT variables.

	Physical Activity
Gender	−0.132
Age	−0.062
Occupation	−0.238 *
Marital status	0.082
Number of children	−0.068
Education	0.081
Income	−0.072
Smoking Status	−0.086
Exercise Self-Efficacy	0.548 ** (0.537 **)
Barrier Self-Efficacy	0.346 ** (0.244 *)
Exercise Social Support (Friends)	0.420 ** (0.336 **)
Exercise Social Support (Family)	0.226 *(0.312 **)
Exercise Social Support	0.372 ** (0.407 **)
Importance of Physical Self	0.303 ** (0.255 *)
Outcome Expectation for Exercise	0.241 * (0.127)
Health Satisfaction	0.318 ** (0.162)
Self-reported Fitness (Strength subscale)	−0.331 ** (−0.382 **)
Self-reported Fitness (Aerobic subscale)	−0.391 ** (−0.421 **)
Self-reported Fitness (Flexibility subscale)	−0.307 ** (−0.337 **)
Self-reported Fitness (Agility subscale)	−0.384 ** (−0.413 **)
Self-reported Fitness (Total)	−0.422 ** (−0.485 **)

Note: ** = *p* < 0.01; * = *p* < 0.05. Partial correlations controlling for demographic variables (gender, age, occupation, marital status, number of children, education, income, and smoking status) were reported in parentheses.

**Table 3:** Hierarchical multiple regression models of predicting PA from demographic variables, SCT measures, and self-reported fitness (n = 77).

	Step 1	Step 2	Step 3
	Coefficient	Coefficient	Coefficient
Age	0.09 (−0.60,.78)	0.30(−0.33,0.93)	0.11(−0.43, 0.66)
Gender	−3.26 (−14.53, 8.01)	7.23 (−4.06, 18.53)	12.58 * (2.75, 22.41)
Occupation	−14.75 * (−27.90, −1.59)	−5.67 (−18.27, 6.93)	−9.63 (−21.10, 1.84)
Self-reported Fitness (Total)		−0.75 ** (−1.11, −0.40)	−0.52 ** (−0.83, −0.21)
Exercise Self-Efficacy			0.29 ** (0.14, 0.43)
Barrier Self-Efficacy			1.59(−3.43, 6.61)
Exercise Social Support Family			0.31(−0.14, 0.75)
Exercise Social Support Friend			0.70 ** (0.19, 1.21)
Importance of Physical Self			0.21(−0.86, 1.28)
Outcome Expectation for Exercise			−3.65(−12.79, 5.48)
Health Satisfaction			−0.03(−0.51, 0.44)
R^2^	0.088	0.270	0.544
△ R^2^	0.088	0.182	0.274
△ F	2.34	17.929 **	5.585 **

Note: 1. Unstandardized coefficients are reported and the 95% confidence intervals are reported in parentheses. 2. The regression was conducted in a stepwise manner. Variables entered in each step were: Step 1, age, gender, and occupation; Step 2, age, gender, occupation, and self-reported fitness (total); Step 3, age, gender, occupation, self-reported fitness (total), and all the SCT variables. 3. ** = *p* < 0.01; * = *p* < 0.05.

## Data Availability

Data are available on request due to privacy restrictions.
